# Nursing students’ understanding of health literacy and health practices: a cross-sectional study at a university in Namibia

**DOI:** 10.1186/s12912-021-00776-z

**Published:** 2022-01-04

**Authors:** Takaedza Munangatire, Nestor Tomas, Violetha Mareka

**Affiliations:** Department of Nursing, University of Namibia, P.O. Box 88, Rundu, Namibia

**Keywords:** Health literacy, Health practices, Nursing students

## Abstract

**Background:**

A poor understanding of health literacy and inadequate health practices among nurses can be detrimental to a population’s health. The starting point of solving this problem is through the provision of methodical health literacy and health practice education in the nursing curriculum. This study explored nursing students’ understanding of the concept of ‘health literacy’ and their health practices at a university in Namibia.

**Methods:**

A descriptive cross-sectional study was carried out among 205 nursing students. A simple stratified sampling method was used and data were collected using a self-reporting Understanding of Health Literacy (UHL) questionnaire. Pearson correlation, independent t-test and One-way ANOVA were used to analyse the data.

**Results:**

The overall mean Understanding of Health Literacy score was 13.04 ± 1.52. The majority (*n* = 157; 76.5%) of the students were found to have adequate health literacy scores, 21.5% had moderate health literacy scores, and only 2% had inadequate health literacy scores. The overall mean health practice score was 32.4 ± 5.50. Most (*n* = 106; 51.7%) of the students were found to have poor health practices, 44.4% had average health practices, and just 3.9% had good health practices. There was no significant relationship between the health literacy levels and health practices of the students (*p* = 0.63).

**Conclusions:**

Nursing students have a good understanding of the concept of health literacy, but more effort can be made to translate this understanding into health literacy skills. There is a need to investigate the contributing factors to poor health practices, as well as develop strategies that can support good health practices among nursing students. These health literacy skills could then be transferred into the students' professional careers as nurses.

**Supplementary Information:**

The online version contains supplementary material available at 10.1186/s12912-021-00776-z.

## Background

Health literacy refers to people’s knowledge, motivation and skills to obtain, comprehend, critique and make use of health information in decision making about healthcare, disease prevention and health promotion, so as to maintain or improve their quality of life [1]. Researchers’ interest in health literacy (HL) has grown rapidly over the years [2], however their focus of late has shifted from assessing HL among the general population to HL among healthcare professionals [3, 4]. Studies show that researchers generally measured healthcare professionals’ HL in a similar way to the general population [5, 6] However, there is a need for health professionals to understand HL from a professional perspective and explore how their understanding relates to their health practices (HP). The emphasis of healthcare professionals’ education should be on how to enhance and increase their understanding of HL, because many studies have demonstrated that nursing professionals do not possess the required levels of HL [7–9].

HL is important for a number of reasons. The first of these is that people’s decisions about health are made based on their HL levels [10], i.e. individuals with good HL levels have a better understanding of their health and are more likely to experience good health outcomes [11]. The health outcomes of individuals are more influenced by their HL than by any other determinants of health [12]. Low levels of HL are thus associated with poor self-care and quality of life [13]. In addition, people with low HL levels do little to promote their health, use health services, and adhere to treatments [14, 15]. Health inequalities have been found to be high in communities with poor HL levels [16], with evidence suggesting that people with inadequate HL are more likely to engage in risky health behaviours [17]. Therefore it is important, that efforts be directed towards raising the understanding of HL of healthcare professionals, who in turn have the responsibility of facilitating the development of HL among the general population [18, 19].

HL among nursing students, nurses and the patients is cause for concern for healthcare authorities [1, 12]. Several studies indicate that HL levels are insufficient amongst both nursing students and nurses, thus it is necessary to integrate HL into undergraduate curricula as well as professional development courses [20–22]. In turn the nursing students and nurses have the responsibility for improving patients’ understanding of HL [12, 23]. If nursing professionals fail to understand HL from a professional point of view, this can compromise their role of developing HL among patients. Consequently, nursing education programmes should develop HL skills among nursing students and improve their health practices before graduating [19].

A study that used the Turkish Literacy Scale [24] found that approximately 50% (*N* = 808) of the nursing student sample had adequate to excellent HL levels, and suggested that gender, age and year of education influenced this [25]. In a study that used the Health Literacy Questionnaire, HL increased according to the number of years of study among undergraduate nursing students (*n* = 845) [26].

Additional studies have revealed that the HL levels of nursing students are inadequate [16]. When applying the Health Literacy questionnaire in a study among different groups of university students, it was found that nursing students reported the lowest HL [27]. Similarly, a study that used the Newest Vital Signs questionnaire indicated that only 37.3% (*n* = 337) of the respondents had adequate HL. The study further reported that HL was a predictor of health-related behaviour among the nursing students [17]. By using the Health Literacy Knowledge and Experience Survey (HL-KES), one study showed that 31.35% (*N* = 160) of students had acceptable HL levels, however there was not a significant relationship between the HL scores and the HP of the nursing students, whose HP were reported to be poor [22]. Additionally, HL was found to be highest among nursing students with chronic conditions and those at graduate level [28].

On the contrary, two separate studies reported that nursing students had sufficient HL levels [19, 29]. In an investigation of HL levels using the Adult Health Literacy Scale, the levels were rated high, with the senior nursing students scoring highest (*n* = 303) [19]. When using the Health Literacy Scale - Q16 HL among healthcare and social services university students, it was found that 69% (*n* = 52) of the nursing students had better HL knowledge than, social services students [29]. According to these studies, therefore, the literacy levels of nursing students differs from place to place. In addition, these studies show that the relationship between nursing students’ HL levels and their health practices is inconclusive. Regarding HP, studies have reported moderate [24, 30] to good [31] practices among nursing students.

Over the years, researchers have developed a number of HL instruments, which measure different dimensions and domains of HL among the general population. Some of the most common instruments are summarised here:

- The Rapid Estimate of Adult Literacy in Medicine (REALM) measures reading ability in the adult general population [32].
- The Test of Functional Health Literacy in Adults (TOFHLA) measures the reading and understanding ability of the general population in healthcare settings [33].
- The Newest Vital Sign (NVS) focuses on functional health literacy by measuring patients’ numeracy and reading skills [34].
- The Health Literacy Skills Instrument (HLSI) assesses functional health literacy, including reading, oral communication and internet use [35].

There are additional comprehensive instruments which measure a number of dimensions of HL, including the Health Literacy Questionnaire (HLQ) [36], the European Health Literacy Questionnaire (HLS-EU-Q47) [37], and the HLS-EU-Q16 [38]. These instruments are self-reporting and measure aspects related to accessing, understanding, appraising and applying information related to healthcare, as well as health-related practices. One instrument was specifically developed to measure HL in nurses, i.e. the Nursing Professional Health Literacy (NPHLS). The instrument measures general knowledge questions related to health literacy, perceptions of HL, and barriers to implementing HL techniques [5].

Addressing the problem of poor HL levels globally should start with a nursing education that develops the HL skills of students [6]. Currently not all nursing education programmes include a HL component in their curricula. Without focus on HL education, it is necessary to assess nursing students’ understanding of HL [23]. Students’ understanding of HL is rarely addressed and nurse educators do not measure how their training programmes enhance the understanding of HL among their students [19]. Nursing education in Namibia does not focus on HL as a course or part of any nursing courses, yet evidence shows that incorporating HL into nursing curricula can improve HL among nurses [11, 39].

The purpose of this study was to answer the following research questions:

- What is nursing students’ understanding of the concept of health literacy?
- What are the health practices of nursing students?
- Is there an association between students’ understanding of health literacy and health practices?
- Are there any relationships between sociodemographic variables, health practices, and the understanding of healthy literacy?

## Materials and methods

### Study design and sample

A descriptive cross-sectional study was conducted between March and June 2020 among undergraduate nursing students (year one to four) at a university in Namibia. A simple stratified sampling method was used to select a proportional number of students from each year of study: first year (49) 23.9%; second year (54) 26.3%; third year (56) 27.3% and fourth year (46) 22.45%. The total sample size was 205. With a small population of 366, no sample size was calculated, all students were approached to be part of the study.

### Data collection tool

The students were invited to articipate via a WhatsApp message which contained a link to the questionnaire, which also provided a consent form for them to read. The students were informed that they were tacitly providing consent to participate in the study by completing the questionnaire. The students were further informed in the cove letter that their responses would be populated anonymously and that their confidentiality was assured as the data was only accessible to the reseachers. Reminders were sent to the students to encourage participation. A total of 205 students participated out of 366, giving a response rate of 56%.

This study measured the nursing students’ understanding of the concept of health literacy, as well as health practices. The questionnaire had three sections, the first of which asked for sociodemographic variables, i.e. age, gender, marital status, source of income, prior qualification, year of study and whether they had a chronic disease or not. The second section included questions that measured the students’ self-reported understanding of the concept of health literacy, using 16 items with yes/no responses. The last section of the questionnaire measured the nursing students’ health practices using 11 items on a 5-point Likert scale, rated from 1 = never to 5 = always. The constructs ‘understanding of health literacy’ and ‘health practices’ were measured using items developed systematically by the researchers, as they could not find any suitable existing instrument. According to research [39], an instrument should be validated for a specific population and purpose.

The researchers developed their self-administered questionnaire (Understanding of Health Literacy) based on extensive literature that included the definitions of health literacy and existing health literacy tools [1, 5, 6, 32–34, 37, 38]. From the existing literacy tools only those aspects measuring understanding of health literacy were extracted.

To ensure validity, three public health specialists checked the questionnaire items against the objectives of the study, providing comments on wording, clarity and alignment with variables of measurement. The questionnaire was then pre-tested by ten students via interviews to check for clarity of content and the structuring of the questionnaire. Some items were either removed or restructured to improve clarity based on the feedback received. Clarity was further enhanced by presenting the questionnaire in the English language, as all the participants were nursing students and all nursing courses in Namibia are delivered in English. To ensure reliability, the validated questionnaire was administered twice in a two-week interval to a group of nursing students, who were not included in the final study.

Exploratory Factor Analysis can be used to establish the construct validity of a questionnaire [40]. The results of this study met the criteria of construct validity of the purpose of this study and the population. Factor analysis was applied for the dimensions ‘understanding health literacy’ and ‘health practice’ separately.

### Understanding health literacy questionnaire

The items that made up this section were those with eigenvalues greater than one from the EFA results. Kaiser-Meyer-Olkin was used as criteria for assessing adequacy of the sample and suitability for EFA. The KMO value was 0.704, which exceeded the acceptable threshold of 0.5 [41]. Bartlett’s test of sphericity was statistically significant (*p* < 0.001; Chi-square = 1563.20, df 120), thereby justifying the application of EFA.

Four factors were extracted explaining 66.0% of the total variance. These four factors were made up of four items, measuring the following dimensions: access to health information, understanding health information, evaluating health information and utilising health information. The internal consistency scale had a Cronbach’s alpha coefficient of > 0.76, which was considered sufficient. Overall the questionnaire had a Cronbach’s alpha value of 0.75.

The HL scores were obtained by adding up the items’ scores with a maximum of 16 for HL level and a maximum score of 55 for health practice. The following classification was used to categorise the HL level: adequate literacy level (13–16), moderate literacy level (9–12), and inadequate literacy level (0–8), and for health practices: good health practices (44–55), moderate health practices (33–43), and poor health practices (11–32). The Cronbach’s alpha value for the whole questionnaire was found to be 0.75.

### Data analysis

The researchers analysed the data using the SPSS 26.0 programme. The frequencies, percentages, mean and standard deviation (mean ± SD) were computed as descriptive statistics for age, HL levels and health practices. The Pearson correlation was used to analyse the relationship between age and HL levels and health practices, while an independent t-test was used to determine the differences in HL levels and health practices for the different groups according to gender, sexual activity and chronic disease status. A one-way ANOVA test was used to compare the mean HL level and health practices scores for the four different groups based on their year of study.

## Results

### Sample characteristics

The mean age of the participants was 22.73 (± 5.69), with males making up 55% and females 45% of the participants. The majority were single (92.8%) and 64.9% of the sample reported they were sexually active. As undergraduate students, a large proportion of the participants (92.9%) received financial support from their parents. Almost as many as (89.9%) had no prior tertiary level qualification. The composition of the participants per year of study was 23.9% first year students, 26.3% second year students, 27.3% third year students and 22.45% fourth year students.

### Health literacy

The mean HL scores were 12.53, 12.98, 13.54 and 13.07 for first, second, third and fourth year students respectively (see Table 1), while the mean health practice scores were 31.6, 31.9, 32.7 and 33.2 for first, second, third and fourth year students respectively (see Table 1).

The majority (*n* = 157; 76.5%) of the students were found to have adequate HL scores, while 21.5% had moderate HL scores and only 2% had inadequate HL scores (see Table 2). Most (*n* = 106; 51.7%) of the students were found to have poor health practices, 44.4% had average health practices, and 3.9% had good health practices (see Table 3).

**Table 1:** Health Literacy and Health Practice Scores

	HL Scores			HP Scores		
**Year of study**	**n (%)**	**Mean**	**SD**	**n (%)**	**Mean**	**SD**
First year	49 (23.9)	12.53	1.79	49 (23.9)	31.6	5.13
Secondyear	54 (26.3)	12.98	1.49	54 (26.3)	31.9	5.67
Third year	56 (27.3)	13.54	1.18	56 (27.3)	32.7	5.05
Fourth year	46 (22.45)	13.07	1.45	46 (22.45)	33.2	6.16
**Total**	**205 (100)**	**13.04**	**1.52**	**205 (100)**	**32.4**	**5.50**

**Table 2:** Health Literacy Score Categories

HL Score Category	n	%
Inadequate (0–8)	4	2.0
Moderate (9–12)	44	21.5
Adequate (13–16)	157	76.5
**Total**	**205**	**100**

**Table 3:** Health Practices Score Categories

Health practices category	n	%
Poor	106	51.7
Average	91	44.4
Good	8	3.9
**Total**	**205**	**100**

### Correlations

There was no significant relationship between the HL levels and health practices of the students (r = 0.033, *p* = 0638). The Pearson correlations showed that there was no significant correlation between the HL of the students and their age (r = 0.091, *p* = 0.196), while the Pearson correlation between health practice and age was significant with a low positive relationship (r = 0.364, *p* = 0.000).

### Independent t-tests

No significant differences were found between the HL scores for the male and female groups, the sexually active and non-sexually active groups, and the chronic and non-chronic disease groups (see Table 4).

The independent t-tests showed that there was a significant difference between the mean scores of female and male participants on health practices (*p* = 0.000) CI [5.2, 2.318], as well as a significant difference between the mean scores of the sexually active group and the non-sexually active group (*p* = 000), CI [1.823, 4.933] (see Table 4).

**Table 4:** Independent t-test for HL and health practices among the groups

Health literacy						
	**Mean(SD)**		**p value**		**Confidence Intervals**	
**Gender**						
Male	13.21(1.40)		0.161		-0.72,0.12	
Female	12.91(1.59)					
**Sexual activity**						
Sexually active	13.05(1.53)		0.873		-0.41,0.48	
Not sexually active	13.01(1.50)					
**Chronic disease status**						
Chronic disease	13.68(1.11)		0.747		-2.18,-3.03	
No chronic disease	12.98(1.54)					
**Health practice**						
	**Mean(SD)**		**p value**		**Confidence Intervals**	
**Gender**						
Male		30.72(4.81)		0.000		-5.20,-2.32
Female		34.48(5.64)				
**Sexual activity**						
Sexually active		33.45(4.95)		0.000		1.82,4.93
Not sexually active		30.07(5.92)				
**Chronic disease status**						
Chronic disease		32.79(6.16)		0.055		-0.02,-1.42
No chronic disease		32.36(5.42)				

### One-way ANOVA

The ANOVA for the four groups showed that there was no significant difference between the mean health practice scores, but there was a significant difference between the mean HL levels (*p* = 0.008) (see Table 5).

**Table 5:** ANOVA on HL and health practice scores among the different groups of nursing students

Health literacy			
**Level of study**	**Mean(SD)**	**95% Confidence Interval for Mean**	**p value**
**First year**	12.53 (1.79)	12.02,13.05	0.008
** s year**	12.98 (1.49)	12.58,13.39	
**Third year**	13.54 (1.18)	13.22,13.85	
**Fourth year**	13.07 (1.45)	12.63,13.50	
**Total**	**13.04 (1.51)**	**12.84,13.25**	
**Health practices**			
**Level of study**	**Mean(SD)**	**95% Confidence Interval for Mean**	**p value**
First year	31.61 (5.14)	30.14,33.09	0.462
s year	31.93 (5.69)	30.37,33.48	
Third year	32.71 (5.05)	31.36,34.07	
Fourth year	33.22 (6.16)	31.39,35.05	
**Total**	**32.36 (5.50)**		

## Discussion

### Health literacy level

This study is one of the few that have reported on nursing students’ understanding of the concept of HL and their HP; most studies reported on the HL of nursing students in a similar way to the general population. We argue that nursing students and healthcare professionals ’understanding of concept HL is different from their HL levels measured from a patient’s perspective.

The study found that the overall understanding of the concept of HL was moderate (21.5%) to adequate (76.5%) (*n* = 205), i.e. nursing students understand the concept of HL more than they possess HL skills. Prior studies reported that 69% (*n* = 52) [31] and 50% (*n* = 808) [27] of nursing students had adequate HL. Even lower levels of HL have been reported, at only 41.7% (*n* = 283) [46], 31.35% (*n* = 160) [19] and 37.3% (*n* = 337) of nursing students having adequate HL. This study shows that nursing students have a good understanding of the concept of HL, suggesting that the nursing curriculum in Namibia could be adequately addressing the concept.

### Health practices

The low HP scores in aspects such as diet, exercise and health seeking behaviours are in contrast to previous studies’ findings, which showed that the HP of students were moderate to high [30, 42]. In addition other studies reported high HP (86.6% of the students had fair to good nutritional habits) [31] and other students had average score of 7.18 on a HP scale measuring from 0 to 10 (0 being poor health and 10 excellent) [31]. These differences could be attributed to context and cultural lifestyles.

### Relationship between health literacy and health practices

In this study, there was no significant correlation between understanding HL and HP. The understanding HL scores were generally high, whereas the HP scores were generally poor. This finding suggests that nursing students need not only understand the HL as professionals, but they also need to develop HL skills for their own use. Existingtant studies reported a significant relationship between HL and HP [17, 22], i.e. people with poor HL have poor health practices, such as a lack of health promotion and non-adherence to treatments [14]. This supports the argument in this study that there is a difference between understanding HL as a health professional, and possessing HL skills as an individual. Nursing students can thus understand HL and help patients develop HL and HL skills.

### Determinants of health literacy and health practice

An analysis of the characteristics of the nursing students and their understanding of HL showed that only age was significantly correlated to the HL scores. Gender, marital status and chronic disease status were not correlated to HL. Previous studies confirm that age significantly influences HL [28], but neither gender nor having a chronic disease determine the level of HL [28, 43]. However, gender has been found to show differences in HL levels among nursing students [25], and students with chronic diseases showed higher levels of HL compared to those without [19]. The current results should be interpreted with caution as it is not known yet if understanding HL as a healthcare professional is the same as possessing HL skills as an individual.

### Health literacy per year of study

This study showed that there was no significant difference (*p *> 0.05) in the average literacy level scores from first year to fourth year nursing students. These results are inconsistent with other reports indicating that senior nursing students had significantly higher mean HL scores compared to first year nursing students (*p* < 0.05) [10]. Furthermore, the results were inconsistent with the findings of [26, 43], which showed that the difference in HL mean scores and student years were statistically significant. The lack of specific HL content in the nursing curriculum, which leaves the development of HL knowledge to chance, could explain the differences between our findings and literature.

### Health practice per year of study

The study results reported that health practices were not significantly different across the four years of nursing education, which is in contrast to previous studies that found that final year nursing students showed significantly higher self-reported health practices than first year students [28]. As students progressed in their nursing studies, they showed improved health practices, including getting more exercise and implementing better dietary practices [44]. The lack of emphasis on self-care among nursing students could explain the poor health practices found in our study.

### Implications of the study’s findings

It is important for nursing students to be understand the concept of HL and to attain good HP before completing their studies. Nurse educators should equally monitor their students’ understanding of HL and HP during the nursing education programme to make sure that the curriculum supports the development of the understanding of HL and HP among nursing students. Efforts should be made to ensure that students understand the importance of HL and HP for themselves as well as their future patients when they become professional nurses. It will take a comprehensive and deliberate approach to include HL and HP in the curriculum and make it part of the major assessments.

## Strengths and limitations

The strength of this study is that it showed the effects of the current nursing curriculum in Namibia on the student nurses’ understanding of HL and on their HP. This study measured nursing students’ understanding of HL rather their HL levels as done in most studies. In addition, by comparing the understanding of HL scores with HP, the study highlighted that the understanding of HL does not necessarily translate into good HP.

The major limitation of the study is that it was conducted at one satellite university campus; expanding the study to the whole university and other universities in Namibia could strengthen the arguments presented in this study. Furthermore, the study was a cross-sectional study; future studies could track the pattern of changes in HL and HP among the students from the time they enter the nursing programme to the time they graduate.

## Conclusion

Nursing students have good understanding of HL, but more effort should be applied to maintain their understanding during and beyond the point of graduation. Regarding poor HP, there is a need to further investigate the contributing factors for this and develop curriculum strategies that can support good HP among nursing students. The good understanding of HL of nursing students is not supported by good HP which can send conflicting messages to their patients. This can make it difficult for the nursing students to transfer of HL skills to patients. As age was found to be positively associated with HP determinant of health practices, it is important to support young students to adopt good health practices, as well as investigate what is it about age that makes people more health conscious as they grow older.

## Supplementary information

**Additional file 1**

## Data Availability

The datasets generated and/or analysed during the current study are not publicly available due to participant privacy, but are available from the corresponding author upon reasonable request and approval by the School of Nursing Ethical Committee of the University of Namibia.
