# Supplementary material for: Nursing students’ understanding of health literacy and health practices: a cross-sectional study at a university in Namibia
Source: BMC Nurs. 2022 Jan 4;21:8. doi: 10.1186/s12912-021-00776-z (PMC8725331; doi:10.1186/s12912-021-00776-z)
Supplement: Supplementary file 1 — Additional file 1 [file 12912_2021_776_MOESM1_ESM.docx]

**UNDERSTANDING HEALTH LITERACY QUESTIONNAIRE**

**SECTION A: DEMOGRAPHIC DATA**

1. What is your gender?

Male Female  Other

1. What is your age?
2. What is your marital status?

Married Single Other

1. Are you sexually active?

Yes No

1. What is your source of income?

Parents  Sponsor Part-time employment

1. What is your prior nursing qualification?

None Certificate Diploma

1. What is your level of study?

First year Second year Third year Fourth year

1. Are you living with any chronic disease?

Yes No

**SECTION B: HEALTH LITERACY**

**Understanding health information**

1. Health literacy is the ability to understand treatment prescriptions

Yes☐ No☐

1. Health literacy is the ability to understand health diagnosis information

Yes☐ No☐

1. Health literacy is the ability to understand medical test results

Yes☐ No☐

1. Health literacy is the ability to understand written health education information.

Yes☐ No☐

**Evaluating health information**

1. Health literacy means one has the ability to select appropriate health-related information.

Yes☐ No☐

1. Health literacy means one has the ability to evaluate the usefulness of health information.

Yes☐ No☐

1. Health literacy means one has the ability to make an informed health care decision.

Yes☐ No☐

1. Health literacy means one has the ability to interpret medical test results.

**Access to health information**

1. Health literacy means being able to regularly search for health information.

Yes☐ No☐

1. Health literacy means having the motivation to search for health information.

Yes☐ No☐

1. Health literacy means being able to regularly access health information.

Yes☐ No☐

1. Health literacy means being able to consult health care providers when in doubt.

Yes☐ No☐

**Utilising health information**

1. Health literacy means one is able to seek health care promptly in response to any changes in health status.

Yes☐ No☐

1. Health literacy means having the ability to correctly carry out health care orders outside the health facility.

Yes☐ No☐

1. Health literacy means one has the ability to comply with treatment.

Yes☐ No☐

1. Health literacy means one has the ability to recognise changes in their health status.

Yes☐ No☐

**SECTION C: HEALTH PRACTICE**

1. How often do you eat a balanced diet?

Always Most of the time Sometimes Rarely Never

1. How often do you smoke?

Always☐ Most of the time☐ Sometimes☐ Rarely☐ Never☐

1. How often do you drink alcohol?

Always☐ Most of the time☐ Sometimes☐ Rarely☐ Never☐

1. How often do you sleep at least 8 hours a night?

Always☐ Most of the time☐ Sometimes☐ Rarely☐ Never☐

1. How often do you seek help when you are stressed?

Always☐ Most of the time☐ Sometimes☐ Rarely☐ Never☐

1. How often do you exercise?

Always☐ Most of the time☐ Sometimes☐ Rarely☐ Never☐

1. How often do you use a condom if you are having sex?

Always☐ Most of the time☐ Sometimes☐ Rarely☐ Never☐

1. How often do you share health-related information?

Always☐ Most of the time☐ Sometimes☐ Rarely☐ Never☐

1. How often do you complete your prescribed treatment?

Always☐ Most of the time☐ Sometimes☐ Rarely☐ Never☐

1. How often do you seek health care if you feel unwell?

Always☐ Most of the time☐ Sometimes☐ Rarely☐ Never☐

1. Do you go yearly routine medical check-up?

Always☐ Most of the time☐ Sometimes☐ Rarely☐ Never
